# Accumulated dose to the rectum, measured using dose–volume histograms and dose-surface maps, is different from planned dose in all patients treated with radiotherapy for prostate cancer

**DOI:** 10.1259/bjr.20150243

**Published:** 2015-08-11

**Authors:** Jessica E Scaife, Simon J Thomas, Karl Harrison, Marina Romanchikova, Michael P F Sutcliffe, Julia R Forman, Amy M Bates, Raj Jena, M Andrew Parker, Neil G Burnet

**Affiliations:** ^1^ Cancer Research UK VoxTox Research Group, Department of Oncology, University of Cambridge, Addenbrooke's Hospital, Cambridge, UK; ^2^ Department of Oncology, University of Cambridge, Cambridge Biomedical Campus, Addenbrooke's Hospital, Cambridge, UK; ^3^ Medical Physics Department, Cambridge University Hospitals NHS Foundation Trust, Cambridge, UK; ^4^ Department of Physics, University of Cambridge, Cavendish Laboratory, Cambridge, UK; ^5^ Department of Engineering, University of Cambridge, Cambridge, UK; ^6^ Cambridge Clinical Trials Unit, Cambridge University Hospitals NHS Foundation Trust, Cambridge, UK

## Abstract

**Objective:**

We sought to calculate accumulated dose (*D*_A_) to the rectum in patients treated with radiotherapy for prostate cancer. We were particularly interested in whether dose–surface maps (DSMs) provide additional information to dose–volume histograms (DVHs).

**Methods:**

Manual rectal contours were obtained for kilovoltage and daily megavoltage CT scans for 10 participants from the VoxTox study (380 scans). Daily delivered dose recalculation was performed using a ray-tracing algorithm. Delivered DVHs were summated to create accumulated DVHs. The rectum was considered as a cylinder, cut and unfolded to produce daily delivered DSMs; these were summated to produce accumulated DSMs.

**Results:**

Accumulated dose-volumes were different from planned in all participants. For one participant, all *D*_A_ levels were higher and all volumes were larger than planned. For four participants, all *D*_A_ levels were lower and all volumes were smaller than planned. For each of these four participants, ≥1% of pixels on the accumulated DSM received ≥5 Gy more than had been planned.

**Conclusion:**

Differences between accumulated and planned dose-volumes were seen in all participants. DSMs were able to identify differences between *D*_A_ and planned dose that could not be appreciated from the DVHs. Further work is needed to extract the dose data embedded in the DSMs. These will be correlated with toxicity as part of the VoxTox Programme.

**Advances in knowledge:**

DSMs are able to identify differences between *D*_A_ and planned dose that cannot be appreciated from DVHs alone and should be incorporated into future studies investigating links between *D*_A_ and toxicity.

## INTRODUCTION

Prostate cancer is the most common cancer affecting males in the UK, accounting for 26% of all new cases of cancer in males in England in 2012.^1^ Overall survival measured at 10 years from diagnosis is the fourth highest of all cancers in the UK at 84%.^2,3^ External beam radiotherapy (RT) is one of several treatment options available to patients with non-metastatic disease.^4^ The introduction of three-dimensional (3D) conformal RT in the 1990s reduced toxicity and so enabled dose escalation to the prostate; this translated into improved tumour control as measured by biochemical progression in various clinical trials.^5–9^ This was, however, at the expense of increased ≥grade 2 late gastrointestinal toxicity, seen in each trial.^7–11^

Since this time, development of other technologies, such as intensity modulation and image guidance, has enabled the dose to normal tissues to be reduced, with consequent improvements in toxicity.^12–17^ Advances in dose computation offer the potential to reduce the dose to normal tissues even further, by adapting the dose distribution during treatment on an individual basis. If the dose could be reduced to critical structures then there may be opportunity for further dose escalation to tumour. Methods to estimate this may identify a subgroup of patients in whom safe dose escalation could be undertaken. These approaches are likely to have application in prostate cancer given the positional variation of the rectum during treatment, its proximity to the tumour target and the use of hypo-fractionated schedules.^18–27^

In order to be able to accurately predict normal tissue complication probability (NTCP) in an individual, understanding the precise relationship between dose and toxicity is essential. At present, predictive models are based on “planned” dose–volume histograms (DVHs) calculated from a single planning CT scan before treatment.^28^ This does not incorporate any measure of anatomic variation during treatment, which has been shown to affect dose to the rectum in several studies.^19,23,24,29–31^ In addition, DVHs as produced from the treatment plan are not ideal representations of 3D doses as they discard spatial information.^28^

To our knowledge, four studies in the literature have used daily image guidance CT scans to calculate accumulated dose (*D*_A_) to the rectum over a course of RT for prostate cancer.^23,24,30,31^ Two used helical tomotherapy megavoltage (MV) scans, one used CT-on-rails and one used MV cone-beam CT. Whilst providing useful early data, each of these studies was limited by one or more factors. These included: missing scans (unable to be retrieved), poor quality scans (in some cases the prostate was not scanned and in some there were unacceptable artefacts), inadequacy of imaged rectal volumes, inspection of only one or two dose-volume levels, and deforming planned dose distributions onto new anatomy, rather than recalculating the dose. We sought to improve on these, by recalculating dose on daily scans from patients treated with RT to prostate and pelvic lymph nodes, and summating these data to produce accumulated DVHs. We also developed dose–surface maps (DSMs) as a way of retaining 3D dose information. These measures of *D*_A_ will be correlated with toxicity as part of the VoxTox study.^32^

## METHODS AND MATERIALS

Ethics approval for the VoxTox study was granted on 4 February 2013. This is an observational study to collect comprehensive toxicity data for 1500 patients undergoing image-guided intensity-modulated radiotherapy (IMRT) to the prostate, head and neck and central nervous system. The aim is to establish the toxicity following treatment and how this relates to the *D*_A_ to the rectum, salivary glands and hypothalamic–pituitary axis.

Rectal contours for 10 study participants were obtained as previously described.^18^ Each participant was treated using helical tomotherapy, with intensity modulation and daily MV image guidance and positional correction to a dose of 74 Gy in 37 fractions over 7.5 weeks.^33^ Following training with a radiologist, JES outlined the rectum on the kilovoltage (kV) planning scan and daily MV image guidance scans for the 10 participants using ProSoma^®^ (Oncology Systems Limited, Shropshire, UK). The entire circumference of the rectal wall was contoured on each slice of each MV scan where it was shown. Digital imaging and communications in medicine (DICOM) RT plans, and the translational and rotational setup errors (X, Y, Z and roll) identified by the radiographers for each fraction, were retrieved from the TomoTherapy^®^ (Accuray, Sunnyvale, CA) archive using in-house software.^34^ All data were anonymized and tokenized and then transferred from the hospital network to the University Physics Laboratory for storage, curation and processing.

Analysis of the MV CT scans revealed that the external contour of the body laterally was not shown in some cases. This was due to the scan circle of the MV CT being 38.6 cm diameter, and too small to encompass the entire patient outline. To address this, the relevant daily shift was applied to the centre of the MV CT, and the corresponding part of the kV image was used for any tissue falling outside the circle. A masked kV image was created by setting all Hounsfield unit (HU) values within the circle to −1000 (corresponding to zero density), and merged with the MV CT. The superior rectum was present on all MV scans; in cases where the MV scan did not extend sufficiently to show the inferior rectum, contours and slices from the kV scan were used.^18^

A dose calculation programme was written as a command-line application in MATLAB^®^ (MathWorks^®^, Natick, MA). This performed a ray-tracing algorithm over a set of points covering the image.^35^ Dose calculations were carried out at each point, using a radiological path length calculated as the sum of those for the MVCT image and for the masked kV image. The use of a separate masked image, rather than one composite image, took account of differences in pixel spacing and HU to electron density conversions between the scans. For MV scans, the HU to electron density conversion varied slightly with the date of image acquisition. We have measured this using a “cheese phantom” with density inserts as part of the monthly quality assurance checks over a seven-year period. The appropriate conversion was used for each MV scan; this depended on the date of the scan and which of our two TomoTherapy machines was used. The calculated dose cube was saved as a DICOM RT dose object. The superior rectum was present on all MV scans; in cases where the MV scan did not extend sufficiently to show the inferior rectum, contours and slices from the kV scan were used.^18^

Daily “delivered” DVHs were produced for each participant; these were summated to produce “accumulated” DVHs. Volumes of rectum receiving 20, 30, 40, 50, 60, 65, 70 and 75 Gy, and doses to 80%, 70%, 65%, 60%, 55%, 50%, 40%, 30%, 25%, 15%, 5% and 3% of the rectum, were compared with those planned. Accumulated generalized equivalent uniform doses (gEUDs) were calculated using *a* = 11.11 as per quantitative analyses of normal tissue effects in the clinic (QUANTEC).^36^ The doses and volumes chosen comprised the constraints from two recent trials.^37,38^

**Figure 1.**
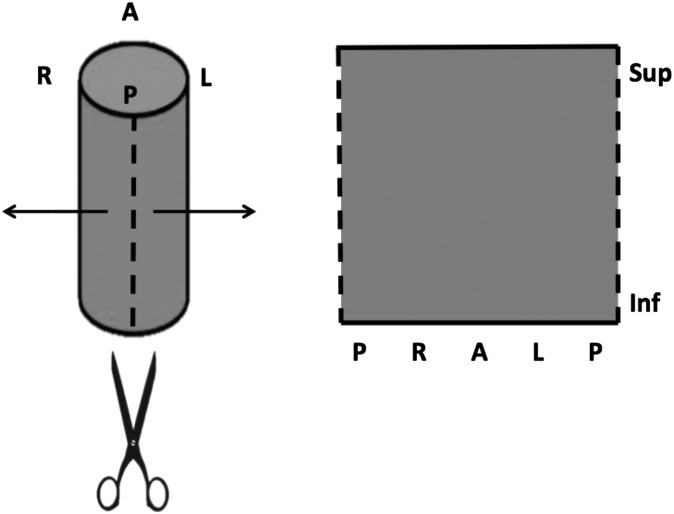
Graphical illustration of the rectum as a cylinder. The cylinder was “cut” at the point where a vertical line from the centroid of each contour crossed the posterior edge, and unfolded. A, anterior; Inf, inferior; L, left; P, posterior; R, right; Sup, superior.

Planned and daily delivered DSMs were produced based on algorithms described by Murray et al^39^ and Buettner et al.^40^ The rectum was considered as a cylinder and dose was sampled at points on each CT slice. The cylinder was “cut” at the point where a vertical line from the centroid of each contour crossed the posterior edge and unfolded (Figure 1).

Each pixel on a DSM comprised a horizontal (posterior–posterior) coordinate and a vertical (superior–inferior) coordinate, with the doses at these points displayed. To obtain the horizontal coordinates, the rectal circumference was measured on the outline for each CT slice and divided by 20. Coordinates were then determined as 21 equally spaced points around the circumference with the first and 21st being at the cut point. For the vertical coordinates, we used the longitudinal coordinates of the kV CT slices. The dose at each of these points was then determined by interpolation into the calculated dose matrix.

**Figure 2.**
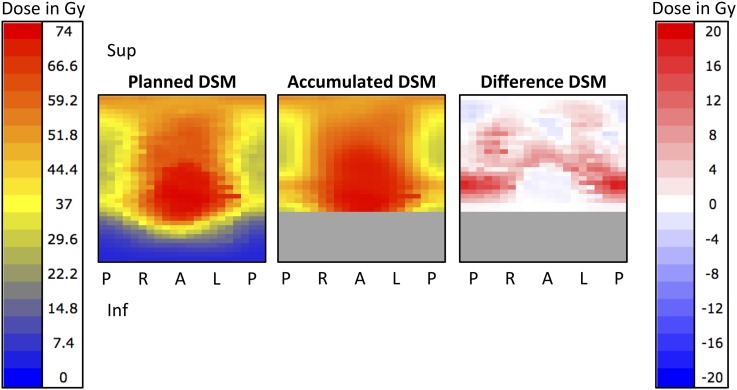
Planned dose–surface map (DSM) and accumulated DSM for Participant A. The difference DSM is also shown, where each pixel from the planned DSM has been subtracted from the equivalent pixel from the accumulated DSM. The dose colour scale for planned and accumulated DSMs is shown on the left and for the difference DSM is shown on the right. Differences in dose of up to +18.2 Gy were seen, particularly to the posterior rectal wall. Where none of the megavoltage scans extended sufficiently inferior, the relevant slices of rectum have been greyed out. A, anterior; Gy, Gray; Inf, inferior; L, left; P, posterior; R, right; Sup, superior.

**Figure 3.**
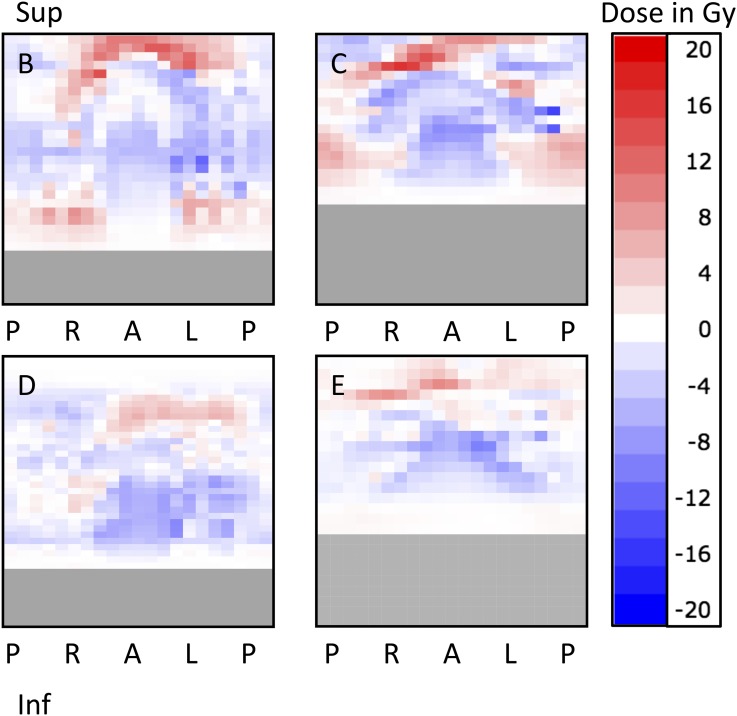
Difference dose–surface maps (DSMs) for Participants B–E. In all four, some pixels received higher accumulated dose (*D*_A_) than planned, despite their dose–volume histograms showing lower *D*_A_s and volumes. The percentage of pixels receiving higher *D*_A_ ranged from 27% (D) to 48% (E). The percentage of pixels receiving ≥5 Gy higher dose than planned ranged from 1% (D) to 12% (C). Where none of the megavoltage scans extended sufficiently inferior, the relevant slices of rectum have been greyed out. A, anterior; Gy, Gray; Inf, inferior; L, left; P, posterior; R, right; Sup, superior.

Accumulated DSMs were constructed by summating the pixels from the delivered DSMs. For participants where the inferior rectum was not shown on any MV scans, this part of the accumulated DSM appeared identical to the planned DSM. To avoid this, in Figures 2 and 3 we have greyed out all slices where none of the MV slices extended sufficiently. Each pixel from the planned DSM was then subtracted from the equivalent pixel from the accumulated DSM to produce a “difference” DSM for each participant. These were inspected to assess whether they provided additional information above that from the DVHs.

To facilitate visual comparison of the DSMs, we developed a system to standardize their superior–inferior lengths. The median MV radius during treatment was calculated for each participant as previously described.^18^ The mean of this value and the corresponding kV radius was used to calculate a rectal circumference for each participant. The circumference was then divided into 20 equal parts, each corresponding to the width of a square pixel on the DSM. For each participant, the superior–inferior kV rectal length was divided by the pixel size. The median of the results for the 10 participants was used as the superior–inferior length of the DSMs.

## RESULTS

**Figure 4.**
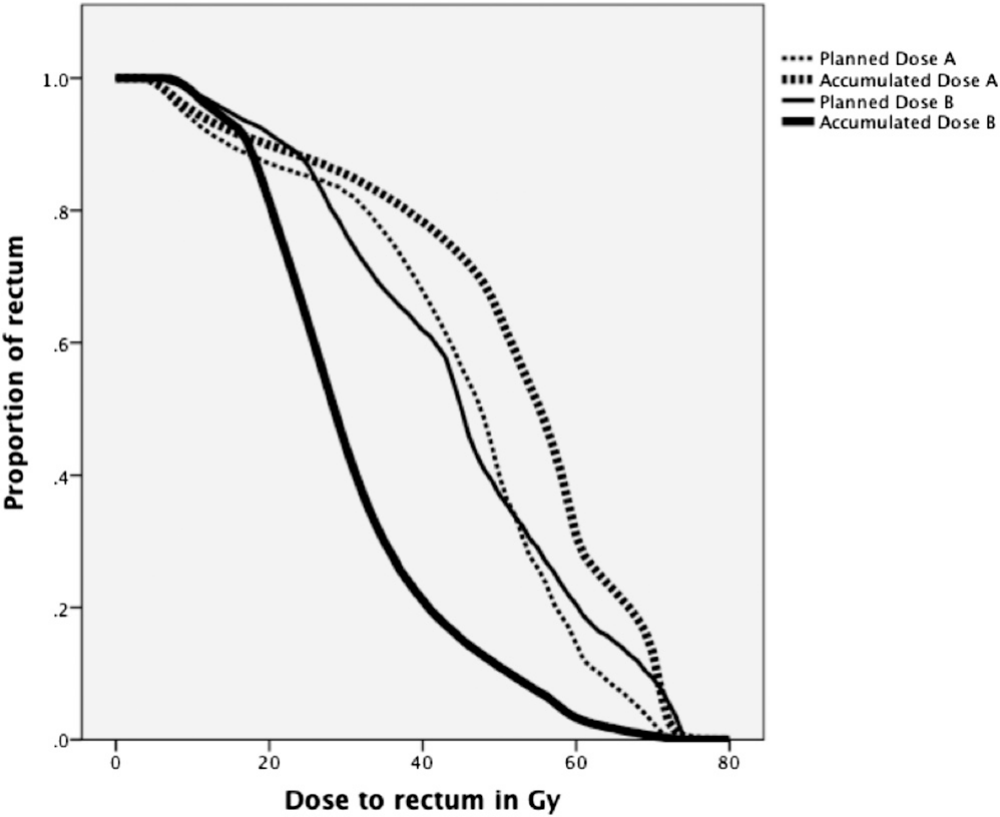
Dose–volume histograms (DVHs) for Participants A and B. The original planned DVHs for both participants were very similar, with planned generalized equivalent uniform dose (gEUD) of 56.9 Gy for A and 60.3 Gy for B. However, the accumulated gEUD for A was 62.2 Gy (5.3 Gy greater than planned) and for B was 50.1 Gy (10.2 Gy less than planned).

The accumulated gEUD was different from the planned gEUD in all 10 participants (median −2.7 Gy, range −10.2 to +5.3 Gy), and lower in 7 of the 10; full results are shown in Table 1. DVHs for the participant with the highest accumulated compared with planned gEUD (Participant A) are shown in Figure 4; DVHs for the participant with the lowest accumulated compared with planned gEUD (Participant B) are also shown (Figure 4). The median relative accumulated gEUD for the 10 participants was 95% of planned (range 83–109%).

**Table 1.** Comparison of planned and accumulated dose (*D*_A_) for the 10 participants using generalized equivalent uniform dose (gEUD). Dose differences (accumulated gEUD minus planned gEUD) are also shown

Participant	gEUD (Gy)		
	Planned	Accumulated	Difference
A	56.9	62.2	5.3
B	60.3	50.1	−10.2
C	58.8	55.8	−3.0
D	53.3	47.3	−6.0
E	57.1	50.2	−6.9
F	57.3	53.8	−3.6
G	60.3	61.3	1.1
H	56.4	55.0	−1.4
I	56.0	53.6	−2.4
J	60.8	61.1	0.3
		Mean	−2.7
		Median	−2.7

**Figure 5.**
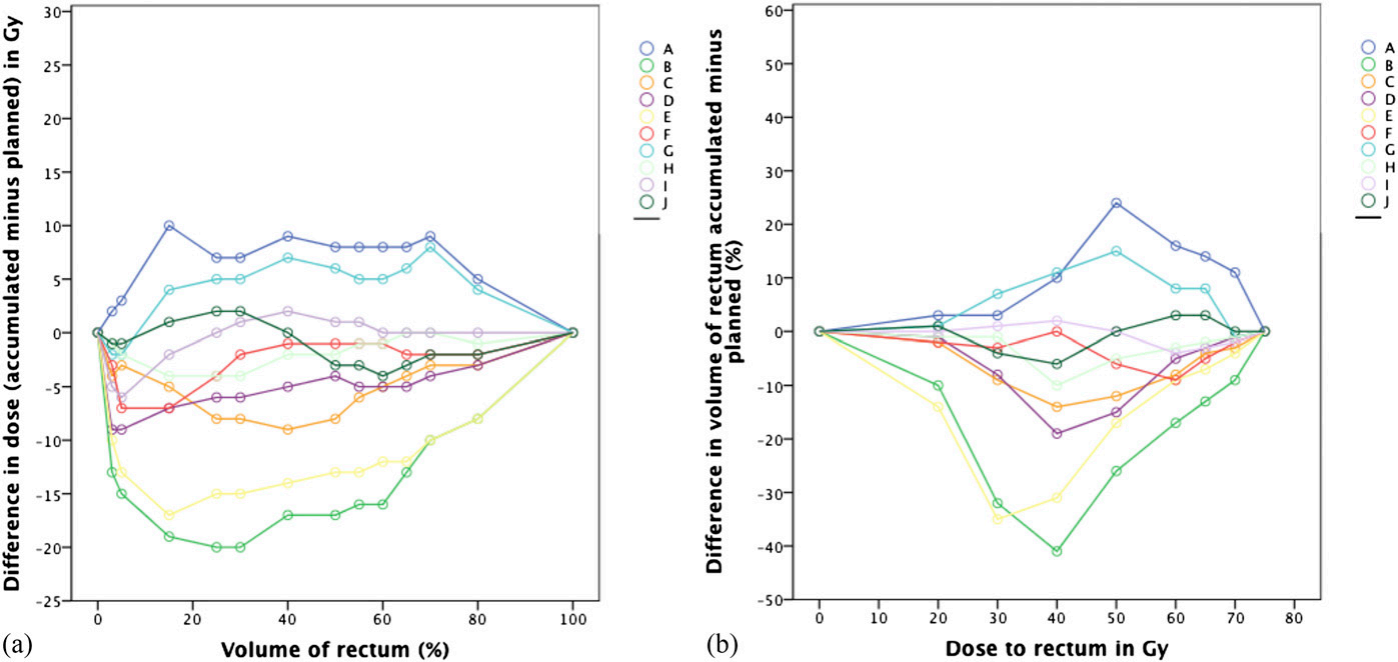
Graphs showing absolute differences in dose (a) and volumes of rectum (b) between accumulated and planned dose–volume histograms (DVHs) for the 10 participants. Doses to 80%, 70%, 65%, 60%, 55%, 50%, 40%, 30%, 25%, 15%, 5% and 3% of the rectum are shown in (a) and volumes treated to 20, 30, 40, 50, 60, 65, 70 and 75 Gy are shown in (b). For Participant A, all accumulated doses and volumes were higher than planned. For Participants B–E, these were all lower than planned.

Differences between accumulated and planned dose to particular volumes of the rectum for the 10 participants are illustrated in Figure 5. Differences in the volumes receiving particular doses are also shown. For one participant (A), all *D*_A_s and volumes were higher than planned. For four participants (B–E), all *D*_A_s and volumes were lower than planned. For one participant (F), all *D*_A_s and volumes were lower or the same as planned. For the remaining four participants, some *D*_A_s and volumes were higher and some lower than planned. Planned, accumulated and difference DSMs for Participant A are shown in Figure 2.

Difference DSMs for Participants B–E are shown in Figure 3. In all four cases, some pixels received higher accumulated than planned dose, despite DVHs showing that *D*_A_s and volumes were lower than planned. The percentage of pixels receiving higher accumulated than planned dose ranged from 27% (D) to 48% (E). The percentage of pixels receiving ≥5 Gy higher accumulated than planned dose ranged from 1% (D) to 12% (C).

## DISCUSSION

Late rectal toxicity occurs ≥90 days after RT and may include bleeding, mucous, urgency, frequent loose bowel movements, pain and incontinence.^36^ These morbidities can be severe and markedly affect the quality of life.^36^ The cumulative incidence of ≥grade 2 toxicity, using the Radiation Therapy Oncology Group (RTOG) scale, in the dose escalated arm of major randomized trials using conformal RT, has been reported as between 18% and 35% with a minimum median follow up of 5.3 years.^7,8,10,11,41^ With IMRT, rates have been reported as between 6% and 26% with a minimum median follow up of 2.4 years.^13–15,27^ Although these data have yet to mature, they indicate that serious toxicity can affect appreciable numbers of males.

Published DVH thresholds for toxicity of RTOG ≥grade 2 converge at doses >70 Gy, and volumes <20%, suggesting that these values are more consistently associated with toxicity.^36^ Five separate fits of the Lyman–Kutcher–Burman NTCP model to rectal toxicity data have estimated similar parameters.^42–46^ The volume effect parameter tended to be small (<0.15), implying serial architecture, with high-dose regions playing the predominant role in determining toxicity risk.^36^ However, other work suggests that volumes receiving lower doses might also be contributory; a constraint for incontinence of *V*_40Gy_ <65–70% has been suggested, for example.^37,47–51^ This may be due to the volumes exposed to intermediate doses playing a role in the recovery of tissue exposed to high doses.^52^ Analysis of the RT01 trial has shown that the number of DVH constraint points which are violated, and the shape of the dose distribution, are correlated with outcome.^51,53^

These models use sample population data to predict the NTCP for an individual based on their RT plan. If a group of patients representative of the underlying population with identical plans was investigated, the median toxicity expressed by the group would be likely to reflect the NTCP. It is highly unlikely that the toxicity expressed by each individual patient would be identical. This is partly explained by variation in intrinsic patient factors reported to influence late toxicity such as diabetes, haemorrhoids, inflammatory bowel disease, advanced age, androgen deprivation therapy, rectum size, prior abdominal surgery and severe acute rectal toxicity.^36^ There is also likely to be a genetic component to the variation; this is an area of active research.^54–56^ The third factor to consider is the difference between the planned DVH and what was actually delivered. This is crucial, as there is potential for monitoring delivered dose during treatment, and for treatment adaptation if required. Minimizing variation in physical dose would also contribute to greater understanding of the role of intrinsic and genetic factors.

In line with the theory that the median toxicity expressed by a group of patients with identical RT plans would equal that predicted, we have shown that for our group of 10 patients, the median position of the axial centre of the rectum over the 370 treatment fractions was within 1 mm of its position at RT planning.^18^ This suggests that at the population level, the RT plan is a good surrogate for *D*_A_. However, considering the participants individually, we found differences between accumulated and planned gEUDs in all 10 cases. 1/10 had higher accumulated than planned gEUD, in 6/10 cases accumulated gEUD was lower than planned and in 3/10 the gEUDs were within 1 Gy of each other. In a group of 8 patients, Akino et al found similar results, with higher accumulated than planned gEUD in 1/8, lower in 6/8 and 1/8 within 1 Gy.^31^

Consistent with this, we also found differences between accumulated and planned DVHs for each of our 10 patients. Accumulated *V*_70Gy_ was higher than planned in one participant (A) and lower by >2% in three participants (B, C and E) as shown in Figure 5. Results were within 2% for the remaining six participants. This is in contrast to previous work by Hatton et al,^29^ where 7/12 patients had higher accumulated *V*_70Gy_ than planned, 3/12 were lower and two were similar. This difference may be due to the frequency of their CT scans being twice a week, rather than daily, and also to their use of cone-beam CT, which may be associated with dose recalculation errors due to uncertainties in the HU values.^29^ 3 out of our 10 participants (30%) had accumulated *V*65_Gy_ > 17%; this is similar to the rate of 27% of scans seen in a study by Chen et al.^19^ Accumulated *D*_3%_ was higher than planned in one participant (A) and lower by >2% in 6/10 participants as shown in Figure 5. In Akino's study, 0/8 patients had higher accumulated *D*_2%_ and it was lower by >2% in 5/8; these are similar to our results.^31^

The most extreme differences in accumulated *V*_70Gy_ were for Participants A and B. Accumulated *V*_70Gy_ for Participant A was 13%, whereas the planned *V*_70Gy_ had been only 3%. For Participant B the reverse was seen; accumulated *V*_70Gy_ was 1%, whereas it was 9% at planning. As doses above this level are more consistently associated with toxicity, we anticipate that Participant A would be more likely to express toxicity than suggested by their NTCP, and that Participant B would be less likely to. If volumes receiving lower doses are also contributory then perhaps Participant G, who received a *V*_40Gy_ of 73% when 62% had been planned, might be more likely to display toxicity than suggested by his NTCP. The converse would be true for Participant E: planned *V*_40Gy_ had been 48%, whereas actually the accumulated *V*_40Gy_ was 17%.

Summated DVHs suffer from a number of limitations when considering *D*_A_. Even when the DVHs on 2 days show the same volume receiving a given dose, the lack of spatial information in a DVH means that these may correspond to different parts of the rectum, meaning that these do not necessarily correspond to a true *D*_A_ DVH. Secondly, a DVH also cannot distinguish between the rectal wall and the rectal contents. The use of rectal wall DSMs, which preserve spatial information, avoids both these problems.

We have successfully implemented a method to generate planned, accumulated and difference DSMs. By “cutting” the surface at a point posterior of the centroid of each image, we ensured that the central column of the DSM corresponded to the middle of the anterior surface of the rectum. This is in contrast to the splitting at the most posterior point of each outline as described by Murray et al, which we found led to large variations from slice to slice. All 10 participants had pixels on the difference DSM that were >+2 Gy; these tended to be located superiorly and posteriorly. This is not surprising, as these areas are away from the prostate, the region where set-up is verified and is consistent with findings from Murray et al.^39^ All 10 participants also had pixels on the difference DSM that were <2 Gy; these tended to affect the anterior rectum. Further work is needed to extract the dose data embedded in the DSMs; approaches that may be relevant include mapping of dose to height and width parameters, eccentricity of higher dose regions and regularity of isodose contours.^53,57^

Previous work with planned DSMs found that the risk of rectal bleeding was higher for patients in whom >37.4% of the pixels received at least 51 Gy.^53^ None of our 10 participants exceeded this threshold on the planned DSM; 2, however, did exceed it on the accumulated DSM (A and J). Although this parameter could have been predicted from the accumulated DVH for Participant A, it would not have been detected for J (Figure 5). Increased bleeding risk has also been found where the lateral extent of the 61 Gy isodose exceeded 59% of the circumference of the rectum.^53^ This was the case for 4/10 of our Participants, A, E, F and J, and applied to both planned and accumulated DSMs. This increased risk for E, F and J would not have been identified from the DVHs. These initial results support the notion that there is additional benefit from incorporating DSMs into dose-toxicity analyses.

A limitation of this work was the lack of availability of images and contours for the most inferior part of the rectum, owing to this being located away from the planning target volume and therefore not scanned for image guidance purposes. Some data were missing for 9/10 participants, with a median of four missing slices per scan (6-mm slice thickness). Fortunately, this part of the rectum is known to have little variation in size and shape.^21,58^ Where shown, contouring of this area was challenging: the superior aspect of the pelvic muscles provided a level below which the wall of the rectum could not be clearly seen on the MV CT scans. We have previously investigated interobserver contouring of this part of the rectum using deliberately extended image guidance scans.^59^ We found that median Jaccard conformity index above the superior pelvic muscles was 0.84, interquartile range (IQR) 0.80–0.87. The corresponding value below the muscles was 0.62, IQR 0.53–0.72. Using independent-samples Kruskal–Wallis test, there was a significant difference between upper and lower portions of the rectum (*p* < 0.001). We suggest that deforming the kV planning outlines onto the MV scans is a pragmatic solution for obtaining lower rectal contours. This will be our strategy in the future where MV slices are unavailable for dose recalculation.

To test whether NTCP based on *D*_A_ is more accurate than with planned dose, large numbers of participants are required, with RT planning, *D*_A,_ and detailed late toxicity data. VoxTox has been set up with this in mind, with anticipated recruitment of 1200 participants treated for prostate cancer. We are developing automated software for contouring and will assess the added value of incorporating voxel tracking using a biomechanical modelling approach. Baseline data will capture intrinsic patient factors and all participants will be offered entry into the Radiogenomics: Assessment of Polymorphisms for Predicting the Effects of Radiotherapy (RAPPER) study, which is assessing polymorphisms for predicting the effects of RT.^56^ We plan to develop NTCP models that incorporate *D*_A_ as a co-variate in addition to planned dose; in the future, intrinsic factors and radiogenomics may become parameters to add to the model.

We propose that this strategy to calculate *D*_A_ would enable more accurate NTCP prediction for each individual undergoing RT for prostate cancer. The delivered dose per pixel could be calculated in real time, with updating of NTCP during the treatment course. For those cases where accumulating dose is suggestive of high NTCP, adaptive re-planning could be undertaken to reduce this to normal levels. This approach would have benefited 1/10 of our participants; if our group is indeed a good estimate of the population then re-planning 10% of patients in clinical practice would be feasible. In cases, where the rectal dose has already been planned to be as low as achievable without compromising target volume coverage, the re-planning could inform decisions on the costs and benefits of reducing the prescribed dose. Conversely, where low NTCP was anticipated, there might be potential for safe dose escalation to tumour. 4/10 of our participants had lower accumulated dose-volumes than those planned and potentially could have tolerated dose escalation to tumour without increasing their NTCP.

## CONCLUSION

Accumulated dose-volumes were different from those planned in 10/10 participants, as measured by gEUD and constraints from recent trials. DSMs provided information additional to the DVH data: four participants had lower *D*_A_s and volumes than planned, but each had pixels on the DSM that received ≥5 Gy more than was planned. Further work is needed to extract the dose data embedded in the DSMs. These will be correlated with toxicity as part of the VoxTox Programme.
